# Blending transcranial direct current stimulations and physical exercise to maximize cognitive improvement

**DOI:** 10.3389/fpsyg.2015.00678

**Published:** 2015-05-22

**Authors:** David Moreau, Chun-Hao Wang, Philip Tseng, Chi-Hung Juan

**Affiliations:** ^1^Centre for Brain Research, University of AucklandAuckland, New Zealand; ^2^Institute of Physical Education, Health and Leisure Studies, National Cheng Kung UniversityTainan, Taiwan; ^3^Graduate Institute of Humanities in Medicine, Taipei Medical UniversityTaipei, Taiwan; ^4^Brain and Consciousness Research Center, Taipei Medical University-Shuang Ho HospitalNew Taipei City, Taiwan; ^5^Institute of Cognitive Neuroscience, National Central UniversityZhongli, Taiwan; ^6^Brain Research Center, National Central UniversityZhongli, Taiwan

**Keywords:** cognitive training, cognitive enhancement, physical exercise, sports training, tDCS, transcranial stimulation, neuroplasticity

## Enhancing cognition: contrasting perspectives, converging applications

Can you make yourself smarter? For over a century, cognitive enhancement has been an elusive endeavor in cognitive psychology. Training on a particular task meant specific, hardly-transferable, improvements (e.g., Ericsson et al., 1993). Recent findings, however, provide reasons to believe that some interventions can induce more general gains. For example, training programs based on physical exercise have shown remarkable benefits (Hillman et al., 2008), in line with recommendations for ecological interventions (Moreau and Conway, 2014). Other non-invasive interventions also appear to be promising—not only to experimental purposes, but also in terms of direct applications to clinical and non-clinical populations (e.g., mindfulness/meditation techniques, interactions with natural environments, cognitive training, brain stimulations). In particular, research based on transcranial Direct Current Stimulation (tDCS) has shown encouraging findings. Here, we address the promise of combining propitious interventions such as tDCS and physical exercise to maximize cognitive improvement.

Before we delve into the means to maximize cognitive enhancement, let us rationalize our idea of combining interventions. The field of cognitive training has been at the core of fairly heated debates in the past few years; for example, what should count as evidence for the effectiveness of an intervention has brought much disagreement (see for example Boot et al., 2013). In theoretical approaches of cognition, the experimental group is usually compared with an active control group—a control group that engages in an equally challenging and interesting training condition, only not in the ability targeted (e.g., Redick et al., 2013). This is often difficult in practice and is source of differences in opinion over what is an adequate control for a given training condition, especially to limit the influence of potential confounding variables (e.g., motivation, Duckworth et al., 2011). Conversely, in clinical settings a novel treatment is usually compared with the best current treatment—what needs to be established in the superiority of the novel intervention over the traditional approach. Such studies are less informative in terms of theoretical understanding of human cognition, but their application is more direct, as they provide clear directions within ecological environments. Eventually, the effectiveness of an intervention and the understanding of its underlying processes converge as more data and knowledge is accumulated—the gap between what is known to work and how it is known to work closes.

From a theoretical point of view, therefore, combining different interventions often obscures experimental results, as each combination needs to be compared against each single treatment it contains. However, when the goal is to benefit human populations, such combinations can be particularly powerful. For example, this idea has led to training programs bridging together physical and cognitive demands, either separately (Shatil, 2013) or within single interventions (Moreau and Conway, 2014). Here, we discuss the potential of combining tDCS with physical exercise—one of the most reliable and well-documented means to trigger general cognitive improvement—and the extent to which such combination can favor long-term neural changes and durable cognitive improvement.

## Combining transcranial direct current stimulations with physical exercise

The past decade has seen the rise of brain stimulation techniques not only as a way to gain a more accurate understanding of human neural circuitry but also to alter patterns of neural activation. In essence, tDCS is a non-invasive stimulation technique that can either facilitate or suppress brain excitability by applying anodal or cathodal electrical stimulation through surface electrodes (Nitsche et al., 2003). Although, the precise mechanisms at play are not well understood yet (Bestmann et al., 2015), the potential of tDCS for cognitive enhancement has been emphasized in numerous research publications (e.g., Banissy and Muggleton, 2013; Richmond et al., 2014; but see also Davis, 2014).

One of the most effective uses of tDCS might be in combination with other methods of cognitive enhancement. In particular, recent research has shown that cognitive training can improve cognitive task performance (Anguera et al., 2013; Mishra et al., 2014), while further enhancement has been reported when cognitive training is combined with tDCS stimulation (Martin et al., 2014). In addition to cognitive training, others have revealed the utility of tDCS in motor skill learning (Reis et al., 2009; Tecchio et al., 2010; Stagg et al., 2011). For example, Stagg et al. (2011), employing tDCS during an explicit motor learning task, found that anodal stimulation was related to faster learning while cathodal stimulation led to slower learning. Notably, delivery of both types of stimulation before the task produced a disruption of learning speed, whereas administration of anodal stimulations following motor training resulted in motor improvement (Tecchio et al., 2010). These findings thus highlight the importance of timing in tDCS stimulation of the motor cortex, depending on the intended purpose (e.g., acquisition, performance, or consolidation; Banissy and Muggleton, 2013). In addition, this suggests that anodal tDCS modulates cortical excitability associated with motor learning (Stagg et al., 2011), thus emphasizing the potential of tDCS to increase neuroplasticity following motor skill learning (Liebetanz et al., 2002).

Given the promising results of non-invasive brain stimulation in the laboratory, one might ask whether there is any way to further maximize the size and the durability of improvements. We argue that one possible approach is to combine brain stimulation with physical exercise. Cognitive enhancement has recently been associated with sport and exercise experience (Hillman et al., 2008; Yarrow et al., 2009; Alves et al., 2013; Moreau and Conway, 2013). There is increasing evidence that physical exercise can improve brain health and plasticity via a variety of mechanisms from cellular to system levels, such as brain-derived neurotrophic factor (BDNF) and insulin-like growth factor (IGF-1) concentrations (Cotman and Berchtold, 2002), brain volume (Erickson et al., 2011), complexity of neuronal activity (Wang et al., 2014), and white matter integrity (Burzynska et al., 2014). Beyond aerobic fitness, studies have also shown that skill sets that are associated with a specific sport can also modulate cognitive performance (Jacobson and Matthaeus, 2014). For example, tennis players possess better temporal processing than fitness-matched athlete controls (Overney et al., 2008; Wang et al., 2013), badminton players have superior visuo-spatial skills and greater modulations on neural oscillations than naïve controls (Jin et al., 2011; Wang et al., 2015), and wrestlers are better at solving mental rotation problems than runners (Moreau et al., 2012). These findings suggest that beyond the general effect of aerobic fitness on cognition, there also is an additional domain-specific benefit to be gained from sports training (Voss et al., 2010; Moreau and Conway, 2014). Thus, sport can be one type of cognitive training that, over a reasonable amount of time, results in more efficient brain functioning (Yarrow et al., 2009; Voss et al., 2010). Given the known effects of tDCS on motor learning, it appears that, if appropriately utilized (e.g., timing of stimulation), tDCS could maximize the effectiveness of sports interventions, either by aiding cognitive or motor learning (see Table 1).

**Table 1:** **Comparison of the principal neural and behavioral changes following tDCS and physical exercise**.

**Level of observation**	**tDCS**	**Physical exercise**
Neurobiological	Acetylcholine ↑	Growth factors (e.g., BDNF, IGF-1, VEGF) ↑
	GABA ↓	Dopamine ↑
	Dopamine ↑	Glutamate ↑
	Glutamate ↑	Norepinephrine ↑
	Cortical activation ↑	Serotonin ↑
		Brain vascularization ↑
		Neurogenesis ↑
		Synaptogenesis ↑
		Brain structure (e.g., cerebral cortex, hippocampus) ↑
Behavioral	Learning ↑	Attention ↑
	Perception ↑	Inhibition ↑
	Response selection ↑	Visuo-spatial cognition ↑
	Motor ↑	Executive control ↑
	Inhibition ↑	Long-term memory ↑
	Working memory ↑	Working memory ↑
	Error awareness ↑	Academic performance ↑
	Rehabilitation ↑	Daily living ↑
		Stress, Anxiety ↓

## Future prospects and challenges

The impact of a combination of brain stimulations and physical training will ultimately depend on the identification of their precise neural underpinnings. To our knowledge, limited research has investigated the effect of combining physical exercise and brain stimulation, presumably because studies often aim to single out one source of facilitation instead of combining a variety of variables for maximized gain. However, studies have shown that the beneficial effect of aerobic fitness on cognition is most evident in the integrity of white matter (Voss et al., 2013), especially in low-fit elderly adults (Burzynska et al., 2014), a finding compelling with the domain-general effect of aerobic exercise. In contrast, the effect of brain stimulation is regionally specific, limiting its effect to superficial cortical (i.e., gray matter) areas. Since the cognitive benefits of physical exercise and tDCS share little common neural areas, it is plausible that effects from combination of both interventions could be additive. Although, contingent on successfully blending several experimental constraints (e.g., retaining adequate statistical power, despite the inclusion of physical exercise only and tDCS only conditions), this idea represents a very promising area of research, with numerous direct applications.

When considering more complex activities such as sports, predicting the outcome of a combination with tDCS becomes slightly more complicated. This is because the cognitive demands of sustained sports training mostly tap long-term changes in cortical areas, and thus adding tDCS to stimulate the same areas might not produce an additive effect. This rationale is supported by a recent study by Bortoletto et al. (2015), where anodal tDCS facilitated learning only when applied concurrently during a non-learning task, and anodal tDCS actually impaired learning when applied during an active learning task that induced cortical excitability. Similar conclusion can also be drawn from a comparison between low and high performers in a study by Tseng et al. (2012), where the authors reported that only low performers benefited from tDCS. Therefore, if a trainee or athlete has already optimized the activation level of a brain region (or network) and reached a high level of performance, the facilitating effect of tDCS may be minimal. Interestingly, several studies have demonstrated that the effect of tDCS can be greater when applied concurrently *during* the practice session. If we view the practice session as a form of cognitive training, these studies might suggest that there is much to be gained by combining cognitive training with brain stimulation (e.g., Ditye et al., 2012). Note that the two ideas are not mutually exclusive—it is possible that brain stimulation could speed up learning rates in cognitive training, while still lacking the ability to overcome plateauing. This emphasizes the importance of considering individual differences in cognitive training (Jaeggi et al., 2014; Moreau, 2014), to offer potent interventions for everyone.

Moreover, one study recently demonstrated that tDCS-related enhancement in motor learning and temporal processing involves BDNF secretion that regulates synaptic plasticity (Fritsch et al., 2010) and increased neuronal complexity (Liang et al., 2014), both of which have been associated with long-term physical exercise (Erickson et al., 2011; Wang et al., 2014). It might therefore be advantageous to test the effect of sport training combined with tDCS stimulation by observing endocrinological and neurophysiological responses, as both may help uncover additional mechanisms behind cognitive changes. Clearly, these arguments need to be experimentally validated before further claims can be made about the efficacy of combining tDCS and physical exercise. Considering the current lack of definitive findings in this area, studies should take advantage of these technological advances to optimize stimulation parameters and training program for maximized cognitive gains.

## Concluding remarks

We have presented herein our views on combining tDCS with other well-established interventions such as physical exercise. The rationale we advocated goes beyond this particular combination, and others have been presented as valid, such as tDCS with cognitive training (e.g., Martin et al., 2013). However, there are unique advantages in considering a combination with physical exercise rather than with cognitive training, as interventions based on the former do not suffer the skepticism associated with the latter. We believe this field of research is promising and will open venues for numerous applications, including healthy aging. In this endeavor, we should keep in mind that long-term consequences of transcranial stimulations are not well understood—scholars have recently been calling for caution regarding the use of these techniques (Davis, 2014), while others have pointed out that the lack of behavioral, physiological, and computational models undermines their clinical applications (Bestmann et al., 2015). With these current limitations in mind, the promise of careful investigations far outweighs the associated risks, and the potential to treat or alleviate numerous conditions remains exciting. If the field of cognitive enhancement is to make a substantial impact on human lives, it needs to incorporate personalized combinations of the most promising interventions.

### Conflict of interest statement

The authors declare that the research was conducted in the absence of any commercial or financial relationships that could be construed as a potential conflict of interest.
